# In vitro contact force evaluation of robotic magnetic navigation catheters; Head to head comparison of different catheters and different settings

**DOI:** 10.1371/journal.pone.0357588

**Published:** 2026-09-28

**Authors:** Zdenek Starek, Filip Souček, Martin Pešl, Martin Folprecht

**Affiliations:** 1 International Clinical Research Center, St. Anne’s University Hospital Brno, Brno, Czech Republic; 2 First Department of Internal Medicine – Cardioangiology, St. Anne’s University Hospital Brno, Brno, Czech Republic; 3 First Department of Internal Medicine – Cardioangiology, Faculty of Medicine, Masaryk University, Brno, Czech Republic; 4 Department of Biology, Faculty of Medicine, Masaryk University, Brno, Czech Republic; 5 Department of Power Electrical and Electronic Engineering, Faculty of Electrical Engineering and Communication, Brno University of Technology, Brno, Czech Republic; Baruch Padeh Medical Center Poriya, ISRAEL

## Abstract

**Background:**

The Stereotaxis Robotic Magnetic Navigation (RMN) system uses an external magnetic field to navigate catheters inside the heart during arrhythmia ablation. This study compared in vitro maximal contact force (CF) achieved with different irrigated RMN ablation catheters under various conditions.

**Methods:**

An in vitro model was created to measure CF using a high-precision goldsmith scale mounted in a dedicated frame between the permanent magnets of the Stereotaxis RMN system. Four irrigated RMN catheters (Navistar RMT, Celsius RMT, Magnoflush, MAGiC™) were evaluated at three magnetic field intensities (0.10 T, 0.08 T, 0.05 T) and under multiple settings.

**Results:**

The MAGiC™ catheter consistently achieved significantly higher CF across all magnetic field intensities and orientations. At 0.10 T, longitudinal median CF ranged from 13.80 g (±0.10) to 19.80 g (±0.50), approximately 36% higher than the other catheters, which ranged from 7.00 g (±0.50) to 14.50 g (±0.38). In the perpendicular orientation, MAGiC™ ranged from 5.09 g (±0.65) to 10.83 g (±0.89), about 156% higher than the others, which ranged from 1.75 g (±0.18) to 4.22 g (±0.36). Across all catheters, CF increased with higher magnetic field intensity, with greater sheath support and with longitudinal orientation.

**Conclusions:**

The MAGiC™ catheter achieved significantly higher CF than other RMN catheters in both longitudinal and perpendicular orientations. The remaining catheters demonstrated comparable CF performance across all magnetic field intensities. Contact force increased with greater magnetic field strength and enhanced sheath support, and was consistently higher in the longitudinal compared with the perpendicular orientation.

## Introduction

Catheter ablation is widely recognized as the therapy of choice for patients with symptomatic arrhythmias, including supraventricular tachycardias (SVT) [1], as well as complex atrial [2] and ventricular arrhythmias [3]. Radiofrequency catheter ablation (RFA) is the most commonly employed invasive treatment strategy and has consistently demonstrated favorable long-term outcomes [2,4]. Conventional RFA is performed in a point-by-point manner using irrigated ablation catheters operated in power-control mode.

To advance beyond standard manual procedures, the **Stereotaxis Robotic Magnetic Navigation (RMN) system** (Stereotaxis Inc., St. Louis, MO, USA) was developed and introduced as an innovative tool in clinical electrophysiology [5,6]. The system generates a magnetic field using strong external permanent magnets to control catheters with magnet-embedded tips. This robotic approach enables precise catheter manipulation, facilitates mapping and ablation in anatomically challenging sites, and reduces radiation exposure for both patients and medical staff [7].

A critical determinant of lesion size, depth, transmurality, and ultimately procedural efficacy and safety is **contact force (CF)**, defined as the force exerted by the catheter tip on the myocardial surface during ablation [8–11]. Despite its advantages, the Stereotaxis RMN system has been criticized for its limited ability to generate sufficiently high CF, for the absence of titration capability, and for the lack of direct force measurement. To date, only two experimental studies have evaluated CF in robotic magnetic navigation (RMN) catheters. In 2018, Bessière and colleagues [8] published an in vitro evaluation of CF with the only RMN catheter available at that time, using a methodology and obtaining results similar to those of the present study. A Polish group Orczykowski et al. [9,10] independently conducted research of similar focus, evaluating the effect of various magnetic vectors, magnetic field intensities, and catheter extensions on the contact force of RMN catheters. In our comparative work, we evaluated the maximum contact force values achievable with all clinically used RMN catheters across all clinically relevant magnetic field intensities and settings.

The objective of this work was to compare the maximal CF achievable in vitro using all different clinically available irrigated **robotic magnetic navigation ablation catheters** with the Stereotaxis system. Specifically, we aimed to (a) evaluate CF across multiple robotic magnetic irrigated ablation catheters, and (b) assess the influence of varying experimental conditions on measured CF.

## Materials and methods

### Experimental model

As depicted in Fig 1, an experimental model was constructed to measure contact force (CF) in vitro using a specialized sensor positioned between the magnets of the RMN system, without patient involvement. The sensor was adapted from a goldsmith scale (MH-360, 100 g/0.01 g; Tmart, Beijing, China), with a measurement accuracy of 0.01 g. It was mounted in a dedicated frame to record CF according to a predefined protocol. The model was placed on an adjustable laboratory table in the electrophysiology suite (Fig 1), positioned between the two permanent magnets of the Stereotaxis Epoch system (Stereotaxis, St. Louis, MO, USA). The tested catheters were inserted using long introducer sheath, (used clinically to introduce catheters into cardiac chambers); in this experiment, we used the Agilis NxT Steerable Introducers (Abbott, Chicago, IL, USA), commonly used as a transseptal sheath, see Fig 1 for details.

**Fig 1:**
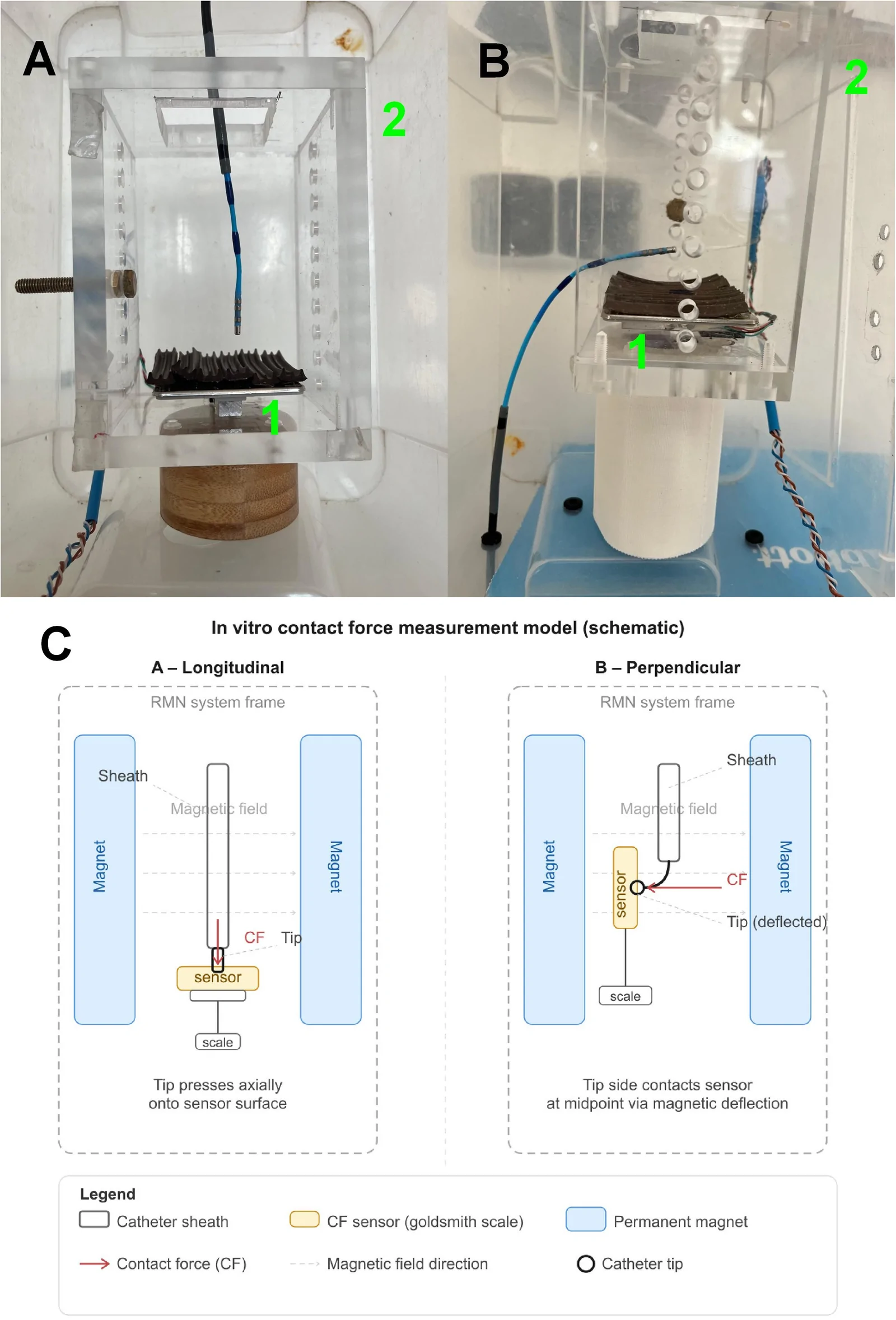
Experimental model. 1 – Measurement sensor, 2 – Plastic frame, A – Longitudinal position of the catheter against the sensor, B – Perpendicular position of the catheter against the sensor. Panel A shows the longitudinal configuration, in which the catheter tip is advanced axially by the QuikCAS™ motor and presses directly onto the sensor surface. Panel B shows the perpendicular configuration, in which the catheter tip is deflected laterally by the external magnetic field and contacts the sensor with its side. The schematic diagram (panel C) illustrates both configurations and the principle of contact force measurement.

Perpendicular catheter movement was generated by the magnetic field of the external magnets, while longitudinal movement was provided by the QuikCAS™ Cardiodrive® sterile device (Stereotaxis, St. Louis, MO, USA) in combination with a specialized servomotor, replacing manual linear advancement of the catheter. The model was designed to mimic catheter–myocardium interaction in two configurations: (I) longitudinal contact, with the catheter tip pressed directly against the wall/sensor, and (II) perpendicular contact, with the side of the catheter tip pressed against the wall/sensor.

### Contact force measurements

Four clinically available irrigated RMN catheters were tested: Navistar RMT, 8 Fr RF ablation catheter (Navistar Thermocool RMT, Biosense Webster, Irvine, CA, USA); Celsius RMT, 8 Fr RF ablation catheter (Celsius Thermocool RMT, Biosense Webster, Irvine, CA, USA); Magnoflush (MedFact Engineering GmbH, Lörrach, Germany); and MAGiC™ (Stereotaxis, St. Louis, MO, USA).

Each catheter was assessed under the following conditions:

- **Magnetic field intensities:** 0.10 T, 0.08 T, and 0.05 T.
- **Contact configuration:** longitudinal (catheter tip against the sensor) and perpendicular (catheter side against the sensor).
- **Irrigation flow rates:** 2 ml/min, 17 ml/min, and 30 ml/min.
- **Sheath support (free end of the catheter protruding from the Agilis sheath)**, Figure 2 in S1 File, see Supplementary materials**:** 3.5 cm, 7 cm, and 14 cm.

For each setting, maximal CF readings were recorded. The scale was recalibrated prior to each measurement and following every measurement the catheter was withdrawn to confirm a baseline value of 0 g, excluding the possibility of electromagnetic interference.

### Statistical analysis

Statistical analyses were performed using R (version 4.2.1) within the RStudio environment. Continuous variables were expressed as median and interquartile range (IQR). The Shapiro–Wilk test was applied to assess the normality of data distribution. Between-group comparisons were performed using Mood’s median test, with post hoc correction according to the Benjamini–Hochberg method.

Results were considered statistically significant when *p* < 0.05 and clinically significant when the absolute difference in medians was ≥ 3 g. Differences were regarded as meaningful only if they were both statistically and clinically significant.

Mood’s median test was selected as the primary between-group comparison method because the Shapiro–Wilk test indicated non-normal distribution in several subgroups. As a non-parametric test designed specifically for comparing medians across independent groups, Mood’s median test is appropriate for the study design and is robust against outliers. The threshold of ≥3 g for clinical significance was chosen based on published data [11] on the minimum contact force associated with lesion formation during radiofrequency catheter ablation, and represents the lower boundary of the 5–25 g range commonly regarded as effective and safe [12] in clinical practice. does not automatically translate

## Results

A total of 2,340 individual contact force (CF) measurements were obtained, yielding 78 median values for statistical analysis. Significant differences were observed between catheters and across experimental conditions. MAGiC™ consistently achieved the highest CF across all magnetic field intensities and orientations.

In the **perpendicular orientation**, median CF for MAGiC™ ranged from 5.09 ± 0.65 g to 10.83 ± 0.89 g, compared with 1.75 ± 0.18 g to 4.22 ± 0.36 g for the other catheters, representing a 156% higher value. In the longitudinal orientation, MAGiC™ ranged from 13.80 ± 0.10 g to 19.80 ± 0.50 g, while Magnoflush ranged from 11.70 ± 0.75 g to 14.50 ± 0.38 g. Both catheters achieved significantly higher values than the remaining catheters (7.00 ± 0.50 g to 10.57 ± 0.50 g), with MAGiC™ demonstrating up to 36% higher CF. Fig 2 displays these comparisons across all three magnetic field intensities for both perpendicular (panel A) and longitudinal (panel B) orientations, with box plots illustrating the median and spread of measurements for each catheter.

**Fig 2:**
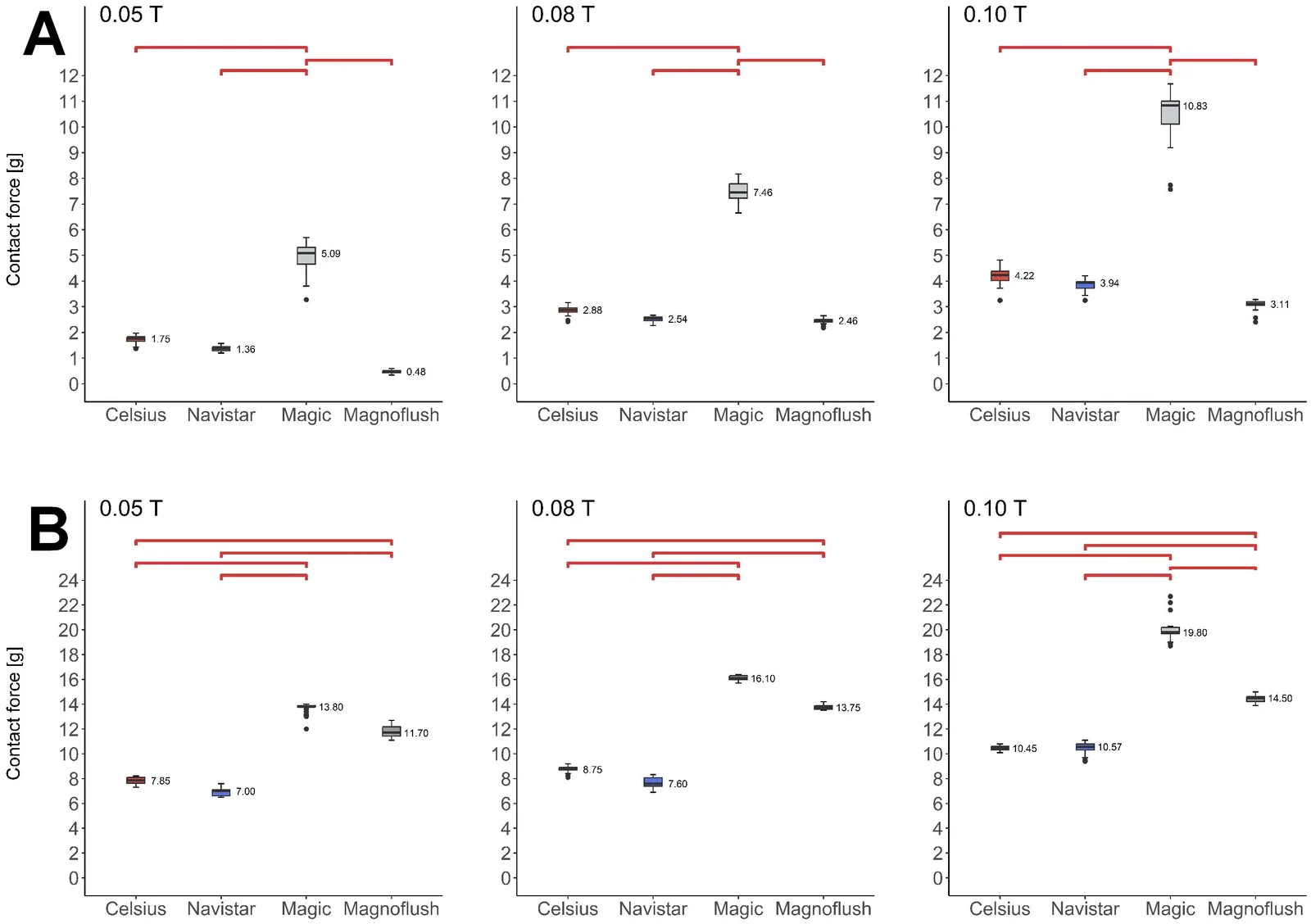
Effect of magnetic field intensity on the maximum achievable contact force for individual catheters at all three magnetic field intensities and both catheter positions. Box plots show median and interquartile range for each catheter at 0.05 T, 0.08 T, and 0.10 T. Red brackets indicate statistically significant differences (p < 0.05 with Benjamini–Hochberg correction). MAGiC™ consistently achieved the highest contact force at all intensities and in both orientations. A – Perpendicular measurement; B – Longitudinal measurement.

Across all catheters, CF increased linearly with higher magnetic field intensity. In the perpendicular orientation, MAGiC™ exhibited both the highest absolute forces and the steepest slope of increase, reaching 10.83 ± 0.89 g. In the longitudinal orientation, the increase was less pronounced, although MAGiC™ again achieved the highest values, up to 19.80 ± 0.50 g. Fig 3 shows this dose–response relationship for each catheter individually, with panel A illustrating perpendicular measurements and panel B longitudinal measurements across all field strengths. Notably, Celsius RMT and Navistar RMT showed a gradual and consistent increase, while MAGiC™ demonstrated the steepest rise, particularly in the perpendicular configuration (Fig 3).

**Fig 3:**
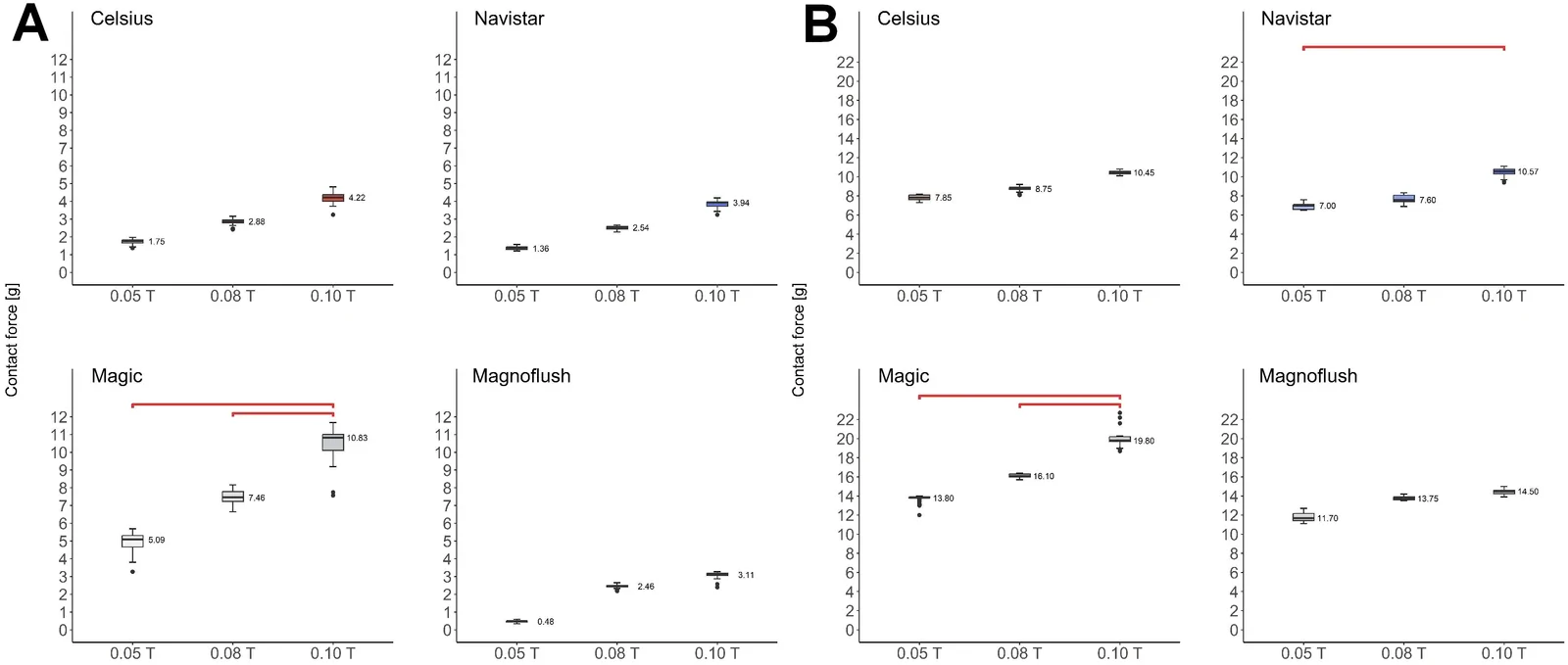
Effect of changing the magnetic field intensity on the maximum achievable contact force for individual catheters and all magnetic field intensities. A – Perpendicular measurement; B – Longitudinal measurement. Each panel shows results for one catheter across all three field strengths (0.05 T, 0.08 T, 0.10 T). Contact force increased progressively with higher magnetic field intensity for all catheters. MAGiC™ demonstrated the steepest increase, particularly in the perpendicular orientation. Box plots show median and interquartile range.

Orientation also played a major role. Longitudinal positioning consistently produced higher CF than perpendicular across all catheters and magnetic field strengths, with Magnoflush showing the largest orientation-related difference (11.39 g at 0.10 T and 3.5 cm sheath support). Fig 4 illustrates this orientation effect for each catheter separately (panels A–D): in every case, the longitudinal values (right box in each subplot) substantially exceeded the perpendicular values (left box), with statistically significant differences at all three field strengths. This pattern was consistent regardless of the degree of sheath support used (Fig 4).

**Fig 4:**
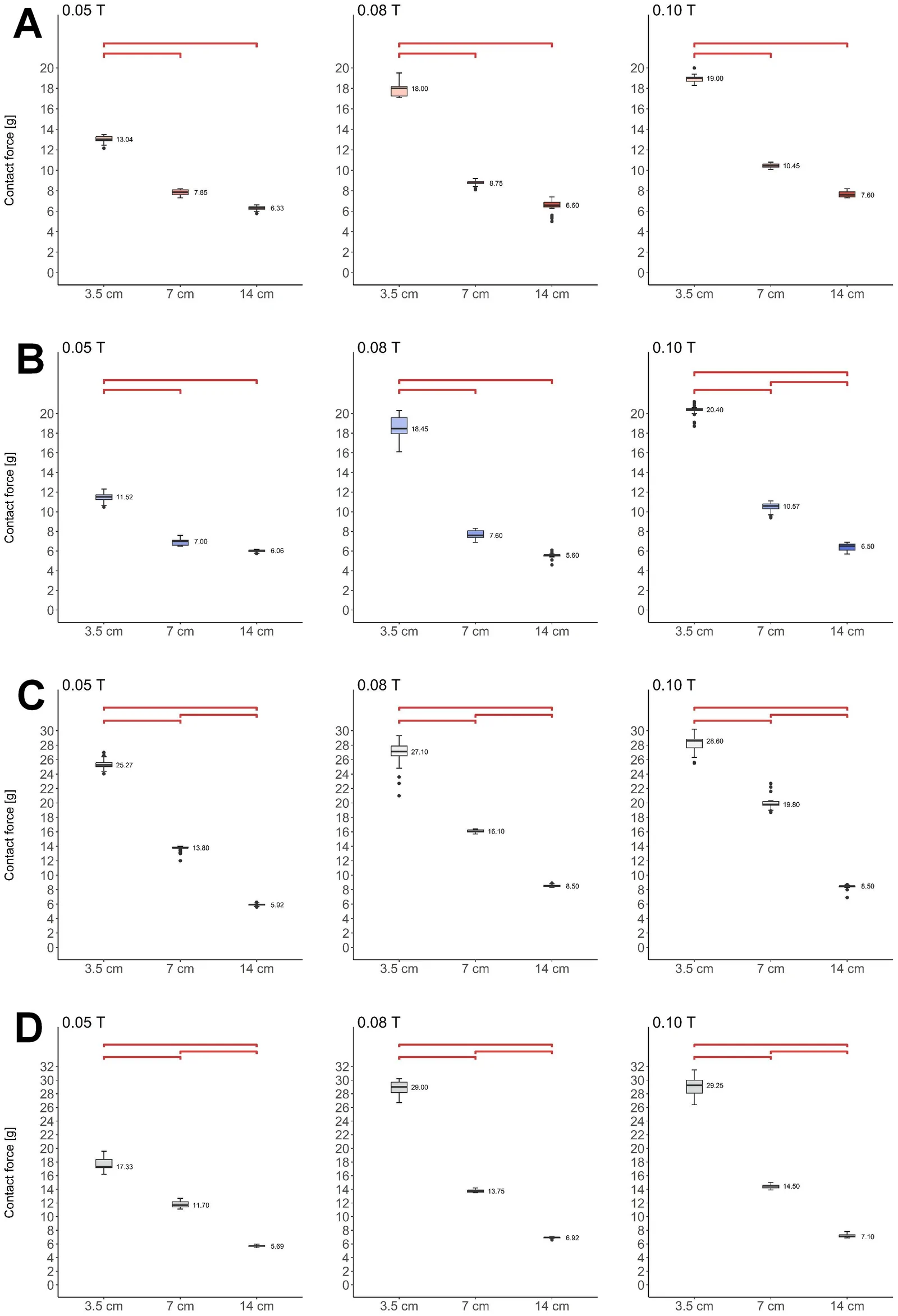
Effect of perpendicular and longitudinal setting on the maximum achievable contact force at different values of magnetic field intensity. A – Celsius; B – Navistar; C – MAGiC™; D – Magnoflush. In all four catheters and at all three magnetic field intensities, longitudinal positioning consistently produced significantly higher contact force than perpendicular positioning (red brackets). The difference was largest for Magnoflush and MAGiC™. Box plots show median and interquartile range; measurements obtained at 3.5 cm sheath support.

Sheath support had a strong effect. Shorter free distal tip lengths consistently produced significantly higher CF across all catheters (Figure 6 in S1 File, see Supplementary materials). As shown in Figure 6 in S1 File, when the free tip was shortened from 14 cm to 3.5 cm, CF approximately doubled for most catheters across all magnetic field intensities, with the effect reaching statistical significance in all comparisons (red brackets). When the combined effects of magnetic field intensity and sheath support were assessed, CF generally increased with higher field strength, although not all differences reached statistical significance. A consistent increase was observed between 0.05 T and 0.08 T for all catheters except MAGiC™. Under these conditions, Magnoflush achieved the highest overall median CF, reaching 29.25 ± 1.90 g at 3.5 cm sheath support and 0.08 T, as shown in Figure 7 in S1 File (see Supplementary materials).

By contrast, irrigation flow rate had no measurable effect. CF values at 2 ml/min, 17 ml/min, and 30 ml/min were statistically comparable in both longitudinal and perpendicular orientations at 0.08 T and 0.10 T. Figure 8 in S1 File (see Supplementary materials) confirms this for all tested catheters and configurations: the box plots for each flow rate are closely overlapping, with no statistically significant differences observed across any catheter or orientation. For technical reasons, this analysis was limited to three catheters (Navistar RMT, MAGiC™, and Magnoflush) and two magnetic field intensities.

## Discussion

To our knowledge, this is the most extensive head-to-head comparative study evaluating contact forces achievable with all currently available irrigated **Robotic Magnetic Navigation (RMN)** electrophysiology catheters. This study represents a comprehensive and sophisticated evaluation of all available RMN catheters, across a wide range of magnetic field strengths and various catheter orientations and extensions.

### Key findings

(A) The MAGiC™ catheter significantly increases contact force while maintaining safety. Across a broad range of settings and parameters, MAGiC™ consistently improves contact force while preserving a strong safety profile. (B) In the Niobe/Genesis 0.08–0.10 T range, the primary limitation of historical catheters was low perpendicular force with results of ~2.5–4.2 grams. MAGiC™ is now 7.5–10.8 grams in these scenarios. The RMN safety profile is preserved, with a maximum observed force of 19.8 grams (longitudinal, 0.10 T). (C) Variability metrics are low, indicating high reproducibility with no outlying values.

Our findings confirm the results of Bessière et al. [8] and Orczykowski et al. [9,10] regarding the effect of magnetic field intensity on contact force. Direct comparison is challenging due to differences in measurement settings; however, the values for individual catheters in both perpendicular and longitudinal settings are comparable to the Bessière data, with and without sheath support.

A substantial difference was observed between perpendicular and longitudinal measurements in all catheters, with contact force at least twice as high in the longitudinal setting. The explanation is straightforward: in the perpendicular setting, contact force is influenced solely by magnetic field intensity. In contrast, during longitudinal measurements, contact force is the combined result of the magnetic field and the longitudinal catheter movement generated by the QuikCAS™ motor.

Our findings regarding sheath support are consistent with previous studies [8–10]. Our results clearly demonstrate a substantial increase in CF with greater sheath support. With a shorter free catheter tip, CF increased to nearly 30 grams.

RMN produces consistent forces within a narrow range. For any given catheter at a specific magnetic field strength, variability is relatively small compared with manual catheters, which exhibit wide fluctuations. RMN catheters, particularly MAGiC™, demonstrate force ranges from perpendicular to longitudinal settings of ~5–9 grams, reflecting stable performance across catheter orientations and cardiac cycle phases.

MAGiC™ in combination with GenesisX performs better than any historical RMN experience. Even at the reduced 0.05 T magnetic field strength of GenesisX, MAGiC™ achieved higher perpendicular forces than any of the historical catheters tested at 0.10 T.

The superior CF performance of MAGiC™ is attributable primarily to its optimized ferromagnetic tip design, which generates a stronger interaction with the external magnetic field and thus greater deflection force. This advantage is evident in both perpendicular and longitudinal configurations. In the longitudinal setting, the QuikCAS™ motor provides controlled catheter advancement and contributes to CF generation; however, since the motor was used identically for all catheters in the longitudinal setting, the relative advantage of MAGiC™ reflects an intrinsic property of its magnetic tip. Future studies comparing CF with the QuikCAS™ system active versus deactivated would help quantify the respective contributions of magnetic tip design and motor-driven advancement.

It is important to note that higher contact force does not automatically translate into improved clinical outcomes [13]. In force-sensing catheter studies, an optimal CF range of approximately 10–40 g has been associated with effective lesion formation, while forces exceeding 40 g carry an increased risk of the steam pops, cardiac perforation and tamponade [12]. In the RMN context, where CF cannot be directly measured or titrated in real time, understanding the range of achievable forces is important for procedural planning and safety. The narrow intra-catheter variability demonstrated in this study suggests that RMN catheters operate consistently within a predictable force range, which may itself be a safety advantage compared with manual catheters, where force fluctuations can be substantially wider.

Based on our findings, several practical recommendations can be made for operators performing RMN-guided catheter ablation. First, the MAGiC™ catheter should be the preferred choice when maximum CF is the primary objective, particularly for ablation at anatomically challenging sites or substrate requiring transmural lesions. Second, higher magnetic field intensity (0.10 T) consistently produced greater CF than lower settings across all catheters; when clinically safe and technically feasible, using the maximum available field strength is preferable. Third, minimizing the length of the free catheter tip (i.e., maximizing sheath support) substantially increases CF and should be exploited whenever anatomy allows. Finally, catheter orientation should be considered: longitudinal contact, achievable with careful catheter positioning, consistently yielded higher CF than perpendicular contact. These parameters can be optimized in combination to maximize lesion quality during RMN-guided procedures.

The emergence of pulse-field ablation (PFA) has introduced a new paradigm for cardiac ablation, characterized by tissue selectivity and reduced risk of collateral injury. However, adequate catheter-tissue contact remains critical for PFA efficacy, as insufficient contact may result in gaps in lesion lines and arrhythmia recurrence. The RMN platform offers several advantages that may remain relevant in the PFA era: reproducible catheter positioning, reduced radiation exposure, and a safety profile associated with low CF variability. The development of RMN-compatible PFA catheters would represent a logical evolution of both technologies. Moreover, RMN retains a unique role in anatomically complex substrates such as ventricular arrhythmias and congenital heart disease, where precise catheter control and the ability to maintain stable contact at challenging sites are paramount. We believe this study provides relevant baseline data that will support ongoing catheter development for the RMN platform.

### Limitations

These in vitro measurements did not account for the influence of blood flow, cardiac motion, or other physiological conditions. Perpendicular and longitudinal models approximate typical catheter–tissue contact patterns, but actual clinical contact represents a combination of both. All measurements were performed in a single magnetic field configuration (sagittal orientation), which is the standard clinical orientation used with the Stereotaxis system. While this limits generalizability to other magnetic vector directions, it reflects the configuration most commonly encountered in clinical practice. The effect of varying magnetic vector orientations on CF has been evaluated by Orczykowski et al. and is outside the scope of this comparative study. Additionally, the experimental model was not validated against a direct force-sensing catheter comparison. Although the scale was calibrated before each measurement and baseline confirmed after each session, future studies should consider validating this type of in vitro model with a contemporary CF-sensing catheter to confirm the accuracy of absolute force values.

## Conclusion

In conclusion, all tested RMN catheters achieved clinically relevant contact forces within a narrow, reproducible range across magnetic field intensities and orientations, indicating consistent performance regardless of catheter approach or simulated cardiac cycle phase. The MAGiC™ catheter demonstrated significantly higher CF in both longitudinal and perpendicular orientations compared with Navistar RMT, Celsius RMT, and Magnoflush, which showed comparable performance to each other. Higher magnetic field strength, greater sheath support, and longitudinal orientation were all associated with increased CF. Irrigation flow rate had no measurable effect on CF.

## Supporting information

S1 File Figure 2. Length of the sheath support – three different lengths of the free catheter tip (distance from the end of the sheath to the catheter tip: 3,5 cm, 7 cm and 14 cm). A shorter free tip (3.5 cm) corresponds to greater sheath support and higher achievable contact force; a longer free tip (14 cm) provides less support and lower contact force. These three configurations were tested for all four catheters across all magnetic field intensities. Figure 6. Effect of different sheath supports on the maximum achievable contact force for individual catheters, A – Celsius; B – Navistar; C – MAGiC™; D – Magnoflush. Shorter free catheter tip length (3.5 cm, left) consistently produced significantly higher contact force compared with 7 cm and 14 cm (red brackets). This effect was observed at all three magnetic field intensities and for all four catheters. Box plots show median and interquartile range. Figure 7. Effect of changing the magnetic field intensity on the maximum achievable contact force for individual sheath support values. Each panel shows results at a fixed free catheter tip length: A – 3.5 cm (maximum sheath support); B – 7 cm; C – 14 cm (minimum sheath support). Within each panel, contact force increased with higher magnetic field intensity for most catheter–sheath combinations. Magnoflush achieved the highest overall contact force at 3.5 cm sheath support (up to 29.25 g). Box plots show median and interquartile range, A – 3,5 cm; B – 7 cm; C – 14 cm. Figure 8. Effect of different flow rates on the maximum achievable contact force at different values of magnetic field intensity. Results are shown for Navistar RMT (panels A–B), MAGiC™ (panels C–D), and Magnoflush (panel E) at 0.08 T and 0.10 T. No statistically significant differences were observed between flow rates of 2, 17, and 30 ml/min in any catheter or orientation, confirming that irrigation flow rate does not influence contact force under the conditions tested. Box plots show median and interquartile range. A – Navistar, perpendicular setting; B – Navistar, longitudinal setting; C – MAGiC™, perpendicular setting; D – MAGiC™, longitudinal setting; E – Magnoflush, longitudinal setting.
(ZIP)
