# Supplementary material for: In vitro contact force evaluation of robotic magnetic navigation catheters; Head to head comparison of different catheters and different settings
Source: PLoS One. 2026 Sep 28;21(9):e0357588. doi: 10.1371/journal.pone.0357588 (PMC13618954; doi:10.1371/journal.pone.0357588)
Supplement: Supplementary file 1 [file pone.0357588.s003.docx]

**Reviewer_com_answer_V5**

**Answer to reviewer comments from 13.7.2026**

1. We note that the grant information you provided in the ‘Funding Information’ and ‘Financial Disclosure’ sections do not match.

Its done, grant specification was moved from Manuscript to the ‘Financial Disclosure’ sections and Cover letter

When you resubmit, please ensure that you provide the correct grant numbers for the awards you received for your study in the ‘Funding Information’ section.

We have registration number CZ.02.1.01/0.0/0.0/16_013/0001826.

a) Please clarify the sources of funding (financial or material support) for your study. List the grants or organizations that supported your study, including funding received from your institution.

Its done, see ‘Financial Disclosure’ section

b) State what role the funders took in the study. If the funders had no role in your study, please state: “The funders had no role in study design, data collection and analysis, decision to publish, or preparation of the manuscript.”

Its done, see ‘Financial Disclosure’ section

c) If any authors received a salary from any of your funders, please state which authors and which funders.

Its done, see ‘Financial Disclosure’ section

Please include your amended statements within your cover letter; we will change the online submission form on your behalf.

Its done, grant specification was moved from Manuscript to the ‘Financial Disclosure’ sections and Cover letter

Please remove any funding-related text from the manuscript and let us know how you would like to update your Funding Statement. Please include your amended statements within your cover letter; we will change the online submission form on your behalf.

Its done, grant specification was moved from Manuscript to the ‘Financial Disclosure’ sections and Cover letter in form „Supported by the state budget through the Ministry of Education, Youth and Sports, project VVI CZECRIN (LM2023049) and by the European Social Fund and the European Regional Development Fund, project CZECRIN_PRO PATIENTS - introduction of innovative modern therapies, reg. number CZ.02.1.01/0.0/0.0/16_013/0001826.“

4. It appears that your ORCiD iD has not been validated in your Editorial Manager account and we are unable to proceed until that step is complete.

Its done,

3. We note that Figures 1 and 2 in your submission contains images which may be copyrighted. All PLOS content is published under the Creative Commons Attribution License (CC BY 4.0), which means that the manuscript, images, and Supporting Information files will be freely available online, and any third party is permitted to access, download, copy, distribute, and use these materials in any way, even commercially, with proper attribution. For more information, see our copyright guidelines: http://journals.plos.org/plosone/s/licenses-and-copyright.

Figures 1 and 2 (fore sure see bellow) are my own work and I think that it is not covered „Creative Commons Attribution License (CC BY 4.0)“ Those figures are only photos of our own catheters and systém and one drawing…

Should I grant copyright to myself?

5. Please ensure that you refer to Table 1 and 2 in your text as, if accepted, production will need this reference to link the reader to the Table.

We do not have any tables in our manuscript, the graphs with the results are marked as Figure 3-6

**Answer to reviewer comments from 23.7.2026**

1. Please upload a copy of Figure 1,2,6,7,8 which you refer to in your text on page 8,9,15. Or if the figure is no longer to be included as part of the submission please remove all reference to it within the text.

Its done

2. We note that there were multiple versions of Manuscript in your submission's file inventory. We have removed the older file(s), retaining the most recent version(s) for editorial review. Please double check your submission file inventory and let us know if any files appear to be outdated or absent.

I checked it and delete older/ outdated versions, sorry I forgott upload answer to reviewer comments for reviwer comments from 13.7., now is uploaded double version – answer from 13.7. comments and answer for 23.7. comments

3. We note that Figures 1 and 2 in your submission contains images which may be copyrighted. All PLOS content is published under the Creative Commons Attribution License (CC BY 4.0), which means that the manuscript, images, and Supporting Information files will be freely available online, and any third party is permitted to access, download, copy, distribute, and use these materials in any way, even commercially, with proper attribution. For more information, see our copyright guidelines: http://journals.plos.org/plosone/s/licenses-and-copyright.

See answer from 13.7.2026 above

We require you to either (1) present written permission from the copyright holder to publish these figures specifically under the CC BY 4.0 license, or (2) remove the figures from your submission:

1. You may seek permission from the original copyright holder of Figures 1 and 2 to publish the content specifically under the CC BY 4.0 license.

We recommend that you contact the original copyright holder with the Content Permission Form (http://journals.plos.org/plosone/s/file?id=7c09/content-permission-form.pdf) and the following text:

“I request permission for the open-access journal PLOS ONE to publish XXX under the Creative Commons Attribution License (CCAL) CC BY 4.0 (http://creativecommons.org/licenses/by/4.0/). Please be aware that this license allows unrestricted use and distribution, even commercially, by third parties. Please reply and provide explicit written permission to publish XXX under a CC BY license and complete the attached form.”

Please upload the completed Content Permission Form or other proof of granted permissions as an "Other" file with your submission.

In the figure caption of the copyrighted figure, please include the following text: “Reprinted from [ref] under a CC BY license, with permission from [name of publisher], original copyright [original copyright year].”

2. If you are unable to obtain permission from the original copyright holder to publish these figures under the CC BY 4.0 license or if the copyright holder’s requirements are incompatible with the CC BY 4.0 license, please either i) remove the figure or ii) supply a replacement figure that complies with the CC BY 4.0 license. Please check copyright information on all replacement figures and update the figure caption with source information. If applicable, please specify in the figure caption text when a figure is similar but not identical to the original image and is therefore for illustrative purposes only.

We've returned your manuscript to your account. Please resolve these issues and resubmit your manuscript within 21 days. If you need more time, please email the journal office at plosone@plos.org. We are happy to grant extensions of up to one month past this due date. If we do not hear from you within 21 days, we will withdraw your manuscript.

Please log on to PLOS Editorial Manager at https://www.editorialmanager.com/pone/ to access your manuscript. You will find your manuscript in the 'Submissions Sent Back to Author' link under the New Submissions menu. Be sure to remove your previous manuscript file if you are uploading a new file in response to these requests. After you've made the changes requested above, please be sure to view and approve the revised PDF after rebuilding the PDF to complete the resubmission process.

We are requesting these changes to comply with the PLOS ONE submission guidelines (https://journals.plos.org/plosone/s/submission-guidelines). Please note that we won't send your manuscript for review until you have resolved the above requests.

Thank you for submitting your work to PLOS ONE and supporting our mission of Open Science.
