# Supplementary material for: In vitro contact force evaluation of robotic magnetic navigation catheters; Head to head comparison of different catheters and different settings
Source: PLoS One. 2026 Sep 28;21(9):e0357588. doi: 10.1371/journal.pone.0357588 (PMC13618954; doi:10.1371/journal.pone.0357588)
Supplement: Supplementary file 2 [file pone.0357588.s001.zip › Supplementary materials.docx]

**Supplementary materials**

**In Vitro Contact Force Evaluation of Robotic Magnetic Navigation Catheters: Head to Head Comparison of Different Catheters and Different Settings**

**Authors**
*Zdenek Starek¹²³, Filip Souček¹²³, Martin Pešl¹²³⁴, Martin Folprecht²

Figure 2. Length of the sheath support – three different lengths of the free catheter tip (distance from the end of the sheath to the catheter tip: 3,5cm, 7cm and 14 cm).

Figure 6. Effect of different sheath supports on the maximum achievable contact force for individual catheters, A – Celsius; B – Navistar; C – MAGiC™; D – Magnoflush.

Figure 7. Effect of changing the magnetic field intensity on the maximum achievable contact force for individual sheath support values, A – 3,5cm; B – 7cm; C – 14cm.

Figure 8. Effect of different flow rates on the maximum achievable contact force at different values of magnetic field intensity. A - Navistar, perpendicular setting; B – Navistar, longitudinal setting; C - MAGiC™, perpendicular setting; D - MAGiC™, longitudinal setting; E – Magnoflush, longitudinal setting.
