# Supplementary material for: In vitro contact force evaluation of robotic magnetic navigation catheters; Head to head comparison of different catheters and different settings
Source: PLoS One. 2026 Sep 28;21(9):e0357588. doi: 10.1371/journal.pone.0357588 (PMC13618954; doi:10.1371/journal.pone.0357588)
Supplement: Peer review history

# Peer review: Decision Letter 0

**DOI:** 10.1371/journal.pone.0357588.r001

**Authors:** Ibrahim Marai

29 May 2026

Dear Dr. Starek,

Please submit your revised manuscript by Jul 13 2026 11:59PM. If you will need more time than this to complete your revisions, please reply to this message or contact the journal office at plosone@plos.org. When you're ready to submit your revision, log on to https://www.editorialmanager.com/pone/ and select the 'Submissions Needing Revision' folder to locate your manuscript file.

- A letter that responds to each point raised by the academic editor and reviewer(s). You should upload this letter as a separate file labeled 'Response to Reviewers'.
- A marked-up copy of your manuscript that highlights changes made to the original version. You should upload this as a separate file labeled 'Revised Manuscript with Track Changes'.
- An unmarked version of your revised paper without tracked changes. You should upload this as a separate file labeled 'Manuscript'.

If you would like to make changes to your financial disclosure, please include your updated statement in your cover letter. Guidelines for resubmitting your figure files are available below the reviewer comments at the end of this letter.

If applicable, we recommend that you deposit your laboratory protocols in protocols.io to enhance the reproducibility of your results. Protocols.io assigns your protocol its own identifier (DOI) so that it can be cited independently in the future. For instructions see: https://journals.plos.org/plosone/s/submission-guidelines#loc-laboratory-protocols. Additionally, PLOS ONE offers an option for publishing peer-reviewed Lab Protocol articles, which describe protocols hosted on protocols.io. Read more information on sharing protocols at https://plos.org/protocols?utm_medium=editorial-email&utm_source=authorletters&utm_campaign=protocols.

As the corresponding author, your ORCID iD is verified in the submission system and will appear in the published article. PLOS supports the use of ORCID, and we encourage all coauthors to register for an ORCID iD and use it as well. Please encourage your coauthors to verify their ORCID iD within the submission system before final acceptance, as unverified ORCID iDs will not appear in the published article. *Only*  the individual author can complete the verification step; PLOS staff *cannot*  verify ORCID iDs on behalf of authors.

We look forward to receiving your revised manuscript.

Kind regards,

Ibrahim Marai, MD

Academic Editor

PLOS One

Journal Requirements:

When submitting your revision, we need you to address these additional requirements.

1. Please ensure that your manuscript meets PLOS ONE's style requirements, including those for file naming. The PLOS ONE style templates can be found at https://journals.plos.org/plosone/s/file?id=wjVg/PLOSOne_formatting_sample_main_body.pdf and https://journals.plos.org/plosone/s/file?id=ba62/PLOSOne_formatting_sample_title_authors_affiliations.pdf

2. We note that the grant information you provided in the ‘Funding Information’ and ‘Financial Disclosure’ sections do not match.

When you resubmit, please ensure that you provide the correct grant numbers for the awards you received for your study in the ‘Funding Information’ section.

3. Thank you for stating the following financial disclosure:

[The author(s) received no specific funding for this work.].

At this time, please address the following queries:

a) Please clarify the sources of funding (financial or material support) for your study. List the grants or organizations that supported your study, including funding received from your institution.

b) State what role the funders took in the study. If the funders had no role in your study, please state: “The funders had no role in study design, data collection and analysis, decision to publish, or preparation of the manuscript.”

c) If any authors received a salary from any of your funders, please state which authors and which funders.

d) If you did not receive any funding for this study, please state: “The authors received no specific funding for this work.”

Please include your amended statements within your cover letter; we will change the online submission form on your behalf.

4. Thank you for stating the following in your manuscript:

[Supported by the state budget through the Ministry of Education, Youth and Sports, project VVI CZECRIN (LM2023049) and by the European Social Fund and the European Regional Development Fund, project CZECRIN_PRO PATIENTS - introduction of innovative modern therapies, reg. number CZ.02.1.01/0.0/0.0/16_013/0001826.]

We note that you have provided funding information that is not currently declared in your Funding Statement. However, funding information should not appear in the Acknowledgments section or other areas of your manuscript. We will only publish funding information present in the Funding Statement section of the online submission form.

Please remove any funding-related text from the manuscript and let us know how you would like to update your Funding Statement. Currently, your Funding Statement reads as follows:

[The author(s) received no specific funding for this work.]

Please include your amended statements within your cover letter; we will change the online submission form on your behalf.

5. We note that you have indicated that there are restrictions to data sharing for this study. For studies involving human research participant data or other sensitive data, we encourage authors to share de-identified or anonymized data. However, when data cannot be publicly shared for ethical reasons, we allow authors to make their data sets available upon request. For information on unacceptable data access restrictions, please see http://journals.plos.org/plosone/s/data-availability#loc-unacceptable-data-access-restrictions.

Before we proceed with your manuscript, please address the following prompts:

a) If there are ethical or legal restrictions on sharing a de-identified data set, please explain them in detail (e.g., data contain potentially identifying or sensitive patient information, data are owned by a third-party organization, etc.) and who has imposed them (e.g., a Research Ethics Committee or Institutional Review Board, etc.). Please also provide contact information for a data access committee, ethics committee, or other institutional body to which data requests may be sent.

b) If there are no restrictions, please upload the minimal anonymized data set necessary to replicate your study findings to a stable, public repository and provide us with the relevant URLs, DOIs, or accession numbers. Please see http://www.bmj.com/content/340/bmj.c181.long for guidelines on how to de-identify and prepare clinical data for publication. For a list of recommended repositories, please see https://journals.plos.org/plosone/s/recommended-repositories. You also have the option of uploading the data as Supporting Information files, but we would recommend depositing data directly to a data repository if possible.

Please update your Data Availability statement in the submission form accordingly.

6. When completing the data availability statement of the submission form, you indicated that you will make your data available on acceptance. We strongly recommend all authors decide on a data sharing plan before acceptance, as the process can be lengthy and hold up publication timelines. Please note that, though access restrictions are acceptable now, your entire data will need to be made freely accessible if your manuscript is accepted for publication. This policy applies to all data except where public deposition would breach compliance with the protocol approved by your research ethics board. If you are unable to adhere to our open data policy, please kindly revise your statement to explain your reasoning and we will seek the editor's input on an exemption. Please be assured that, once you have provided your new statement, the assessment of your exemption will not hold up the peer review process.

7. In the online submission form, you indicated that [Data are available from the corresponding author upon reasonable request. Due to privacy and ethical restrictions, data cannot be made publicly available in compliance with the General Data Protection Regulation (GDPR).].

All PLOS journals now require all data underlying the findings described in their manuscript to be freely available to other researchers, either 1. In a public repository, 2. Within the manuscript itself, or 3. Uploaded as supplementary information.

This policy applies to all data except where public deposition would breach compliance with the protocol approved by your research ethics board. If your data cannot be made publicly available for ethical or legal reasons (e.g., public availability would compromise patient privacy), please explain your reasons on resubmission and your exemption request will be escalated for approval.

8. PLOS requires an ORCID iD for the corresponding author in Editorial Manager on papers submitted after December 6th, 2016. Please ensure that you have an ORCID iD and that it is validated in Editorial Manager. To do this, go to ‘Update my Information’ (in the upper left-hand corner of the main menu), and click on the Fetch/Validate link next to the ORCID field. This will take you to the ORCID site and allow you to create a new iD or authenticate a pre-existing iD in Editorial Manager.

9. We note that Figures 1 and 2 in your submission contains images which may be copyrighted. All PLOS content is published under the Creative Commons Attribution License (CC BY 4.0), which means that the manuscript, images, and Supporting Information files will be freely available online, and any third party is permitted to access, download, copy, distribute, and use these materials in any way, even commercially, with proper attribution. For more information, see our copyright guidelines: http://journals.plos.org/plosone/s/licenses-and-copyright.

We require you to either (1) present written permission from the copyright holder to publish these figures specifically under the CC BY 4.0 license, or (2) remove the figures from your submission:

1. You may seek permission from the original copyright holder of Figures 1 and 2 to publish the content specifically under the CC BY 4.0 license.

We recommend that you contact the original copyright holder with the Content Permission Form (http://journals.plos.org/plosone/s/file?id=7c09/content-permission-form.pdf) and the following text:

“I request permission for the open-access journal PLOS ONE to publish XXX under the Creative Commons Attribution License (CCAL) CC BY 4.0 (http://creativecommons.org/licenses/by/4.0/). Please be aware that this license allows unrestricted use and distribution, even commercially, by third parties. Please reply and provide explicit written permission to publish XXX under a CC BY license and complete the attached form.”

Please upload the completed Content Permission Form or other proof of granted permissions as an "Other" file with your submission.

In the figure caption of the copyrighted figure, please include the following text: “Reprinted from [ref] under a CC BY license, with permission from [name of publisher], original copyright [original copyright year].”

2. If you are unable to obtain permission from the original copyright holder to publish these figures under the CC BY 4.0 license or if the copyright holder’s requirements are incompatible with the CC BY 4.0 license, please either i) remove the figure or ii) supply a replacement figure that complies with the CC BY 4.0 license. Please check copyright information on all replacement figures and update the figure caption with source information. If applicable, please specify in the figure caption text when a figure is similar but not identical to the original image and is therefore for illustrative purposes only.

If the reviewer comments include a recommendation to cite specific previously published works, please review and evaluate these publications to determine whether they are relevant and should be cited. There is no requirement to cite these works unless the editor has indicated otherwise.

[Note: HTML markup is below. Please do not edit.]

Reviewers' comments:

Reviewer's Responses to Questions

**Comments to the Author**

1. Is the manuscript technically sound, and do the data support the conclusions?

Reviewer #1: Yes

Reviewer #2: Yes

**********

2. Has the statistical analysis been performed appropriately and rigorously?

Reviewer #1: I Don't Know

Reviewer #2: Yes

**********

3. Have the authors made all data underlying the findings in their manuscript fully available?

The PLOS Data policy

Reviewer #1: Yes

Reviewer #2: Yes

**********

4. Is the manuscript presented in an intelligible fashion and written in standard English?

Reviewer #1: Yes

Reviewer #2: Yes

**********

Reviewer #1: Dear Authors. Interesting study.

This manuscript presents an in vitro evaluation of contact force (CF) across four robotic magnetic navigation (RMN) ablation catheters under varying magnetic field. The topic is clinically relevant, as CF remains one from important determinant of lesion quality and procedural success in catheter ablation. The authors should be commended for attempting a systematic comparison across multiple catheters and settings.

However, while the study is presented as “comprehensive,” its novelty, methodological rigor, and contextual positioning relative to prior key studies (notably Orczykowski et al. and Bessière et al.) are insufficiently developed. Several important limitations reduce the translational and scientific impact of the work.

Major Comments

The authors claim this is the “first comprehensive assessment,” which is not fully justified.

-The study by Bessière et al. (2018) already demonstrated the relationship between magnetic field strength and CF using a similar in vitro setup.

-Orczykowski et al. (2025) provided a more mechanistic and clinically oriented analysis, including: the impact of magnetic vectors, catheter extension, variability of CF,

It is commendable that the authors included four different RMN catheters, which provides a broader comparative perspective than most prior studies in this field. This aspect clearly strengthens the descriptive value of the work.

However, all measurements were performed in a single magnetic field configuration (sagittal orientation). Given that catheter behavior in RMN is highly dependent on magnetic vector direction, this represents an important limitation. Different vector configurations may substantially alter contact force characteristics and catheter–tissue interaction.

Including additional magnetic field orientations—or at least discussing their potential impact in more detail—would improve the interpretability and generalizability of the findings.

The manuscript introduces a threshold of ≥3 g as “clinically significant,” but this definition is not supported by references or physiological rationale.

Higher CF does not necessarily translate into better clinical outcomes or safety.

The discussion section should cover this topic.

Reporting medians together with “±” values is unconventional. The rationale for using Mood’s median test should be explained.

Minor Comments

Language requires moderate editing for grammar and clarity. (electrophysiology suite??)

1. Some typographical errors and inconsistencies are present.

2. Figures would benefit from more detailed descriptions in the main text.

3. Terminology (e.g., units, abbreviations) should be standardized throughout.

,,For each setting, maximal CF readings were recorded’’- It would be more informative to also report the range of measurements

Line 197

,,According to our knowledge, this is the first comprehensive assessment of the contact forces achieved with Robotic Magnetic Navigation (RMN) electrophysiology catheters.’’

Comment

This is not the first assessment, and it is not comprehensive in every aspect. Please rephrase.

Line 77

,,A critical determinant of lesion size, depth, transmurality, and ultimately procedural efficacy and safety is contact force (CF)’’….

Comment

Previously published data are not as clear regarding whether efficacy is increased.

The conclusion section seems too long.

Suggestions for Improvement

1. Clarify the incremental contribution of the study.

2. Expand discussion on limitations.

3. Moderate conclusions regarding catheter performance.

4. Revise language for clarity and precision.

Reviewer #2: Zdenek Starek et al. investigated “In Vitro Contact Force Evaluation of Robotic Magnetic Navigation Catheters: Head-to-Head Comparison of Different Catheters and Different Settings.”

The Stereotaxis Robotic Magnetic Navigation (RMN) system, which enables catheter manipulation using magnetic navigation without direct manual catheter control, has been used clinically for more than a decade. At a time when the risks of myocardial injury and cardiac tamponade associated with catheter ablation were substantial, the RMN system offered a highly promising safety profile by reducing unintended catheter manipulation and excessive mechanical force on the myocardium. However, several issues, including installation requirements and high costs, have limited its widespread adoption, and the system has not become a mainstream platform for catheter ablation to date.

Around the same period, contact force (CF) catheters rapidly gained popularity, and assessment of catheter tip pressure became increasingly important for achieving effective and reproducible lesion formation. Because the RMN system lacked integrated CF sensing technology, this became a major limitation of the platform. In this regard, the present study is highly interesting and clinically relevant. Moreover, although pulse-field ablation (PFA) has recently emerged, adequate tissue contact remains an important issue, suggesting the potential future relevance of this technology. While the manuscript is well written, several limitations should be addressed.

Concern #1

The current figure does not provide an intuitive understanding of how catheter tip pressure was measured in the experimental setup. A simple schematic diagram illustrating catheter positioning and the method used for CF measurement would improve clarity.

Concern #2

Was any validation experiment performed to confirm that the CF measured in this experimental model approximates actual clinical CF values?

For example, performing the same experimental protocol with a CF-sensing catheter and comparing the measured values would help validate the accuracy and clinical relevance of this experimental model.

Concern #3

An important finding of this study is that sufficient CF could not be achieved with conventional RMN catheters other than the MAGiC catheter. However, the explanation for the superior CF performance observed with the MAGiC catheter remains insufficient. If the authors hypothesize that the QuikCAS™ motor contributed to this effect, additional evidence should be provided. For example, comparison of CF measurements with the QuikCAS™ system activated versus deactivated would help support this conclusion, if technically feasible.

Concern #4

The clinical implications of these findings should be more clearly discussed. Specifically, the authors should clarify whether their results provide practical recommendations for achieving effective lesion formation during RMN-guided ablation. For example, should operators preferentially use higher magnetic field settings in combination with the MAGiC catheter?

Concern #5

Please comment on the future significance of maintaining and developing this technology, even in the current era of pulse-field ablation (PFA).

Concern #6

The conclusion is somewhat lengthy and partially repetitive. A more concise summary focused on the principal findings and clinical implications is recommended.

**********

what does this mean?). If published, this will include your full peer review and any attached files.

If you choose “no”, your identity will remain anonymous but your review may still be made public.

**Do you want your identity to be public for this peer review?**  For information about this choice, including consent withdrawal, please see our Privacy Policy

Reviewer #1: No

Reviewer #2: **Yes:** Yoshifumi Ikeda

**********

[NOTE: If reviewer comments were submitted as an attachment file, they will be attached to this email and accessible via the submission site. Please log into your account, locate the manuscript record, and check for the action link "View Attachments". If this link does not appear, there are no attachment files.]

To ensure your figures meet our technical requirements, please review our figure guidelines: https://journals.plos.org/plosone/s/figures

You may also use PLOS’s free figure tool, NAAS, to help you prepare publication quality figures: https://journals.plos.org/plosone/s/figures#loc-tools-for-figure-preparation.

NAAS will assess whether your figures meet our technical requirements by comparing each figure against our figure specifications.

# Author comment: Author response to Decision Letter 1

**DOI:** 10.1371/journal.pone.0357588.r002

14 Aug 2026

In Vitro Contact Force Evaluation of Robotic Magnetic Navigation Catheters;

Head to Head Comparison of Different Catheters and Different Settings

Response to Reviewers' Comments – Revision 1

We thank the reviewers for their thorough and constructive evaluation of our manuscript. We have carefully addressed all comments. Changes to the manuscript are indicated in the revised text. Below, each reviewer comment is quoted in italics, followed by our response in green, and where applicable, the exact revised manuscript text.

REVIEWER #1

Comment 1 (Major): Novelty claim – "first comprehensive assessment"

The authors claim this is the "first comprehensive assessment," which is not fully justified. The study by Bessière et al. (2018) already demonstrated the relationship between magnetic field strength and CF using a similar in vitro setup. Orczykowski et al. (2025) provided a more mechanistic and clinically oriented analysis, including the impact of magnetic vectors, catheter extension, and variability of CF.

Answer: We agree with this critique and have revised the opening sentence of the Discussion accordingly. We acknowledge the prior contributions of Bessière et al. and Orczykowski et al. and have repositioned our study's contribution as a head-to-head comparative evaluation across all currently available irrigated RMN catheters under a wide range of clinically relevant conditions, which has not been performed before.

Revised manuscript text (Discussion, first sentence): "According to our knowledge, this is the most extensive head-to-head comparative study evaluating contact forces achievable with all currently available irrigated Robotic Magnetic Navigation (RMN) ablation catheters across a wide range of clinically used magnetic field intensities and settings."

Comment 2 (Major): Single magnetic field orientation (sagittal only)

All measurements were performed in a single magnetic field configuration (sagittal orientation). Given that catheter behavior in RMN is highly dependent on magnetic vector direction, this represents an important limitation. Different vector configurations may substantially alter contact force characteristics and catheter–tissue interaction. Including additional magnetic field orientations—or at least discussing their potential impact in more detail—would improve the interpretability and generalizability of the findings.

Answer: We acknowledge this limitation. Our study was designed to compare the maximum achievable CF across all available catheters in the standard clinical sagittal configuration, which represents the most commonly used orientation in clinical practice with the Stereotaxis system. Exploring every possible magnetic vector direction was beyond the scope of this comparative study. We note that Orczykowski et al. (2025) have addressed the effect of different magnetic vector orientations in greater detail. We have expanded the Limitations section of the Discussion to explicitly address this point.

Added to Limitations: "All measurements were performed in a single magnetic field configuration (sagittal orientation), which is the standard clinical orientation used with the Stereotaxis system. While this limits generalizability to other magnetic vector directions, it reflects the configuration most commonly encountered in clinical practice. The effect of varying magnetic vector orientations on CF has been evaluated by Orczykowski et al. [ref] and is outside the scope of this comparative study."

Comment 3 (Major): Clinical significance threshold of ≥3 g – insufficient justification

The manuscript introduces a threshold of ≥3 g as "clinically significant," but this definition is not supported by references or physiological rationale.

Answer: We agree that the threshold requires explicit justification. The 3 g value was chosen based on the established clinical range for effective and safe radiofrequency ablation (5–25 g), as reported in CF catheter studies. A threshold of 1 g is too small to be meaningful, while 5 g is too conservative given the CF levels achievable with RMN catheters. A value of 3 g represents a pragmatic midpoint supported by the authors' clinical electrophysiology experience and is consistent with the lower range of forces associated with lesion formation in prior CF catheter literature. We have added this rationale to the Methods section. We note, however, that this threshold was used only as an additional interpretive criterion alongside the primary statistical significance threshold.

Added to Statistical Analysis section: "The threshold of ≥3 g for clinical significance was chosen based on published data on the minimum contact force associated with lesion formation during radiofrequency catheter ablation [refs], and represents the lower boundary of the 5–25 g range commonly regarded as effective and safe in clinical practice. Values below this threshold were considered insufficient for reliable lesion delivery."

Comment 4 (Major): Higher CF does not necessarily translate into better clinical outcomes or safety

Higher CF does not necessarily translate into better clinical outcomes or safety. The discussion section should cover this topic.

Answer: We agree and have added a paragraph to the Discussion addressing this nuance. While higher CF is generally associated with deeper and more transmural lesions in force-sensing catheter studies, the relationship between CF and clinical efficacy or safety in RMN-guided ablation is more complex, particularly given the absence of integrated CF sensing in current RMN systems. We have also noted the risk of perforation at excessively high CF values and the importance of catheter stability over raw force magnitude.

Added to Discussion: "It is important to note that higher contact force does not automatically translate into improved clinical outcomes. In force-sensing catheter studies, an optimal CF range of approximately 10–40 g has been associated with effective lesion formation, while forces exceeding 40 g carry an increased risk of cardiac perforation and tamponade [refs]. In the RMN context, where CF cannot be directly measured or titrated in real time, understanding the range of achievable forces is important for procedural planning and safety. The narrow intra-catheter variability demonstrated in this study suggests that RMN catheters operate consistently within a predictable force range, which may itself be a safety advantage compared with manual catheters, where force fluctuations can be substantially wider."

Comment 5 (Major): Unconventional reporting of medians with ± values

Reporting medians together with "±" values is unconventional.

Answer: We thank the reviewer for this comment and suggestion. We agree that the "±" notation is unconventional and it is used more with means and SD. We have corrected the notation throughout the manuscript in the following way: median (interquartile range – IQR).

Throughout manuscript: All instances of "median ± value" have been changed to "median (IQR)" format, e.g., "7.85 (1.2) g" instead of "7.85 ± 0.18 g".

Comment 6 (Major): Rationale for Mood's median test not explained

The rationale for using Mood's median test should be explained.

Answer: We have added an explanation to the Statistical Analysis section. Mood's median test was chosen because the Shapiro–Wilk test indicated non-normal distribution in several subgroups and our primary outcome (maximal CF) is expressed as a median. Mood's median test is a non-parametric test, that is appropriate for comparing medians across independent groups. To test the difference, we also needed to take into account the size of the clinically relevant threshold, which was set at a value of 3. Measurements of catheter contact forces differed only if they differed in both statistical and clinical sense.

Added to Statistical Analysis section: "The Shapiro–Wilk test was applied to assess the normality of data. The groups were compared using Mood’s median test, with post hoc correction according to the Benjamini–Hochberg method. To test the difference in this case, it was also necessary to consider the clinically relevant threshold, which was set at a value of 3. To account for statistical difference, the mentioned nonparametric Mood’s median test, which is designed to compare medians of the independent groups of measurements, was used and then to account for clinical difference, it was supplemented with subtraction of the group’s medians. Results were considered statistically significant when p < 0.05 and clinically significant when the absolute difference in medians was ≥ 3 g."

Comment 7 (Minor): Language editing – grammar, clarity, and terminology (e.g., "electrophysiology suite")

Language requires moderate editing for grammar and clarity. (electrophysiology suite??) Some typographical errors and inconsistencies are present. Terminology (e.g., units, abbreviations) should be standardized throughout.

Answer: We thank the reviewer for this comment. The manuscript has been comprehensively reviewed and edited by a native English speaker. Typographical errors and inconsistencies have been corrected. The term "electrophysiology suite" refers to the specialized cardiac catheterization laboratory used for electrophysiology procedures; this is a standard and widely used term in the field. We have retained it but added a brief clarification on first use. Units and abbreviations have been standardized throughout the manuscript. A full list of language corrections is provided in the tracked-changes version of the revised manuscript.

Key language corrections made to the manuscript:

• Introduction, line 77: "A critical determinant of lesion size, depth, transmurality, and ultimately procedural efficacy and safety is contact force (CF)" → revised (see Comment 13 below)

• Methods: "used in clinical practice for introducing catheters into the heart cavities" → "used clinically to introduce catheters into cardiac chambers"

• Methods: "in this experiment we used" → "In this experiment, we used"

• Discussion: "previeous works" → "previous studies"

• Discussion: "According to our knowledge" → "To our knowledge" (used consistently throughout)

• Introduction: "only two experimental study has" → "only two experimental studies have"

• Abbreviations: CF, RMN, IQR, T (Tesla) are now defined on first use and used consistently thereafter.

Comment 8 (Minor): Figures would benefit from more detailed descriptions in the main text

Figures would benefit from more detailed descriptions in the main text.

Answer: We have expanded the figure legends and the in-text descriptions of figures throughout the Results section to provide more detailed context for each figure. Specifically, we have clarified what each panel depicts and highlighted the key findings visible in each figure within the text.

Comment 9: "For each setting, maximal CF readings were recorded" – range of measurements should be reported

"For each setting, maximal CF readings were recorded" – It would be more informative to also report the range of measurements.

Answer: We have added reporting of the minimum, maximum, median and IQR (interquartile range) in the form of tables at the end of the document. For publication, we suggest moving these tables to the section „Supplementary materials“. Additionally, the number of individual measurements per condition is stated clearly in the Methods, and the data are also fully visible in the box plots provided.

Comment 10 (Minor): Line 197: "first comprehensive assessment" – rephrase

"According to our knowledge, this is the first comprehensive assessment of the contact forces achieved with Robotic Magnetic Navigation (RMN) electrophysiology catheters." – This is not the first assessment, and it is not comprehensive in every aspect. Please rephrase.

Answer: Already addressed in Major Comment 1. The sentence has been revised to: "To our knowledge, this is the most extensive head-to-head comparative study evaluating contact forces achievable with all currently available irrigated RMN ablation catheters across a wide range of clinically used magnetic field intensities and settings."

Comment 11 (Minor): Line 77: CF as determinant of "procedural efficacy" – previously published data are not clear

"A critical determinant of lesion size, depth, transmurality, and ultimately procedural efficacy and safety is contact force (CF)" – Previously published data are not as clear regarding whether efficacy is increased.

Answer: We agree. The relationship between CF and procedural efficacy is supported primarily by lesion quality parameters (depth, transmurality, impedance drop) rather than by consistent long-term clinical outcome data. We have revised this sentence to more accurately reflect the current evidence.

Revised (Introduction): "Contact force (CF) – defined as the force exerted by the catheter tip on the myocardial surface – is an important determinant of lesion size and depth, and has been associated with procedural outcomes in radiofrequency catheter ablation [refs]. However, its relationship with long-term clinical efficacy remains incompletely established."

Comment 12 (Minor): The conclusion section seems too long

The conclusion section seems too long.

Answer: We have shortened the Conclusion section by removing repetitive statements and consolidating the principal findings into a more concise summary.

Revised Conclusion: "In conclusion, all tested RMN catheters achieved clinically relevant contact forces within a narrow, reproducible range across magnetic field intensities and orientations, suggesting consistent performance regardless of catheter approach or simulated cardiac cycle phase. The MAGiC™ catheter demonstrated significantly higher CF in both longitudinal and perpendicular orientations compared with Navistar RMT, Celsius RMT, and Magnoflush, which showed comparable performance to each other. Higher magnetic field strength, greater sheath support, and longitudinal orientation were all associated with increased CF. Irrigation flow rate had no measurable effect on CF."

REVIEWER #2 (Yoshifumi Ikeda)

We thank Reviewer #2 for the detailed and thoughtful comments. The reviewer correctly identifies the clinical relevance of this work and raises important questions that we have addressed below.

Comment Concern #1: Lack of intuitive schematic diagram of the experimental setup

The current figure does not provide an intuitive understanding of how catheter tip pressure was measured in the experimental setup. A simple schematic diagram illustrating catheter positioning and the method used for CF measurement would improve clarity.

Answer: We agree that a schematic illustration would substantially improve the reader's understanding of the experimental setup. We have added a schematic diagram to Figure 1 (as a new panel C) that illustrates: (i) the position of the measurement sensor between the permanent magnets, (ii) the longitudinal catheter configuration with the catheter tip contacting the sensor, and (iii) the perpendicular catheter configuration with the catheter side contacting the sensor. The figure legend has been updated accordingly.

Comment Concern #2: No validation experiment comparing measured CF with a CF-sensing catheter

Was any validation experiment performed to confirm that the CF measured in this experimental model approximates actual clinical CF values? For example, performing the same experimental protocol with a CF-sensing catheter and comparing the measured values would help validate the accuracy and clinical relevance of this experimental model.

Answer: This is an excellent and important methodological point. A direct validation with a CF-sensing catheter was not performed in this study, which we acknowledge as a limitation. However, the goldsmith scale used (MH-360, accuracy 0.01 g) was calibrated before each measurement, and baseline confirmation (return to 0 g after catheter withdrawal) was performed after every measurement to exclude electromagnetic interference or scale drift. The values obtained in our model for the historical catheters (Celsius RMT, Navistar RMT) are consistent with those reported by Bessière et al. (2018) using a comparable in vitro methodology, which provides indirect support for the validity of our approach. We have added this as a limitation and suggested validation with a force-sensing catheter as a direction for future work.

Added to Limitations: "The experimental model was not validated against a direct force-sensing catheter comparison. Although the scale was calibrated before each measurement and baseline confirmed after each session, future studies should consider validating this type of in vitro model with a contemporary CF-sensing catheter to confirm the accuracy of absolute force values."

Comment Concern #3: Insufficient explanation for MAGiC™ superior CF – role of QuikCAS™ motor

An important finding of this study is that sufficient CF could not be achieved with conventional RMN catheters other than the MAGiC catheter. However, the explanation for the superior CF performance observed with the MAGiC catheter remains insufficient. If the authors hypothesize that the QuikCAS™ motor contributed to this effect, additional evidence should be provided.

Answer: We thank the reviewer for this important observation. The MAGiC™ catheter's superior CF performance relates primarily to its dedicated magnetic tip design, which contains a stronger ferromagnetic element optimized for the Stereotaxis system and capable of generating greater deflection force under a given external magnetic field. In the longitudinal configuration, the QuikCAS™ motor contributes to CF by providing controlled linear catheter advancement – and this applies equally to all catheters tested in the longitudinal setting. The perpendicular measurements (where the CF difference between MAGiC™ and other catheters is also substantial) are driven entirely by the magnetic field interaction with the catheter tip, without contribution from the motor. This distinction supports the conclusion that the magnetic tip design of MAGiC™ is the primary driver of its superior CF, independent of the QuikCAS™ system. We have clarified this in the Discussion and noted that a direct comparison with QuikCAS™ activated versus deactivated would be a valuable experiment for future studies.

Added to Discussion: "The superior CF performance of MAGiC™ is attributable primarily to its optimized ferromagnetic tip design, which generates a stronger interaction with the external magnetic field and thus greater deflection force. This advantage is evident in both perpendicular and longitudinal configurations. In the longitudinal setting, the QuikCAS™ motor provides controlled catheter advancement and contributes to CF generation; however, since the motor was used identically for all catheters in the longitudinal setting, the relative advantage of MAGiC™ reflects an intrinsic property of its magnetic tip. Future studies comparing CF with the QuikCAS™ system active versus deactivated would help quantify the respective contributions of magnetic tip design and motor-driven advancement."

Comment Concern #4: Clinical implications and practical recommendations for RMN-guided ablation

The clinical implications of these findings should be more clearly discussed. Specifically, the authors should clarify whether their results provide practical recommendations for achieving effective lesion formation during RMN-guided ablation. For example, should operators preferentially use higher magnetic field settings in combination with the MAGiC catheter?

Answer: We thank the reviewer for this suggestion. We have added a paragraph to the Discussion providing explicit practical guidance for operators performing RMN-guided ablation, based on the study findings.

Added to Discussion (Clinical Implications paragraph): "Based on our findings, several practical recommendations can be made for operators performing RMN-guided catheter ablation. First, the MAGiC™ catheter should be the preferred choice when maximum CF is the primary objective, particularly for ablation at anatomically challenging sites or substrate requiring transmural lesions. Second, higher magnetic field intensity (0.10 T) consistently produced greater CF than lower settings across all catheters; when clinically safe and technically feasible, using maximum available field strength is preferable. Third, minimizing the length of the free catheter tip (i.e., maximizing sheath support) substantially increases CF and should be exploited whenever anatomy allows. Finally, catheter orientation should be considered: longitudinal contact, achievable with careful catheter positioning, consistently yielded higher CF than perpendicular contact. These parameters can be optimized in combination to maximize lesion quality during RMN-guided procedures."

Comment Concern #5: Future significance of RMN technology in the era of pulse-field ablation (PFA)

Please comment on the future significance of maintaining and developing this technology, even in the current era of pulse-field ablation (PFA).

Answer: We agree that this is an important contextual question and have added a paragraph addressing the future relevance of RMN technology in light of emerging ablation modalities.

Added to Discussion: "The emergence of pulse-field ablation (PFA) has introduced a new paradigm for cardiac ablation, characterized by tissue selectivity and reduced risk of collateral injury. However, adequate catheter-tissue contact remains critical for PFA efficacy, as insufficient contact may result in gaps in lesion lines and arrhythmia recurrence. The RMN platform offers several advantages that may remain relevant in the PFA era: reproducible catheter positioning, reduced radiation exposure, and a safety profile associated with low CF variability. The development of RMN-compatible PFA catheters would represent a logical evolution of both technologies. Moreover, RMN retains a unique role in anatomically complex substrates such as ventricular arrhythmias and congenital heart disease, where precise catheter control and the ability to maintain stable contact at challenging sites are paramount. We believe this study provides relevant baseline data that will support ongoing catheter development for the RMN platform."

Comment Concern #6: Conclusion is lengthy and partially repetitive

The conclusion is somewhat lengthy and partially repetitive. A more concise summary focused on the principal findings and clinical implications is recommended.

Answer: Already addressed in response to Reviewer #1, Comment 12. The Conclusion section has been substantially shortened and restructured to focus on the principal findings and their clinical implications. Please see the revised text above.

ENGLISH LANGUAGE – FULL CORRECTION SUMMARY

The manuscript has been comprehensively reviewed for language, grammar, and clarity. The following corrections were made (listed by section):

Abstract

• "approximately 36% higher" – kept; phrasing is correct.

• "Rest of catheters demonstrated" → "The remaining catheters demonstrated"

• "Contact force increased with greater magnetic field strength and enhanced sheath support, was consistently higher" → "Contact force increased with greater magnetic field strength and enhanced sheath support, and was consistently higher"

Introduction

• "only two experimental study has evaluated" → "only two experimental studies have evaluated"

• "A Polish group Orczykovski et al." → "A Polish group, Orczykowski et al.," (note: correct spelling is Orczykowski, not Orczykovski)

• "independently engaged in research of a similar focus to ours evaluated the effect" → "independently conducted research of similar focus, evaluating the effect"

• "in our comparative, more comprehensive work" → "In our comparative work" (the claim of comprehensiveness has been moderated; see Comment 1)

• "we compared the effect of all clinically used magnetic field intensities for all clinically used RMN catheters and for all these catheters we compared the maximum contact force values in different clinical settings" → "we compared the maximum contact force values achievable with all clinically used RMN catheters across all clinically relevant magnetic field intensities and settings"

Methods

• "used in clinical practice for introducing catheters into the heart cavities" → "used clinically to introduce catheters into cardiac chambers"

• "in this experiment we used" → "In this experiment, we used" (comma added, sentence capitalized)

• "Figure 2, see Supplementary materials" → "(Figure 2; see Supplementary Materials)" (consistent formatting)

• "Faculty od Electrical Engineering" → "Faculty of Electrical Engineering" (typo corrected in affiliations)

Results

• Median ± IQR notation changed to median [IQR] throughout (see Comment 5)

• "yielding 78 median values for statistical analysis" – kept; accurate description.

• "significantly higher CF across all magnetic field intensities and orientations" – correct; no change.

Discussion

• "According to our knowledge" → "To our knowledge" (standard English phrase; corrected consistently throughout)

• "previeous works" → "previous studies"

• "Our findings regarding sheath support are consistent with previeous works" → "Our findings regarding sheath support are consistent with previous studies"

• "force ranges from perpendicular to longitudinal settings of ~5–9 grams" → "force differences between perpendicular and longitudinal settings of approximately 5–9 g"

• Grams: standardized to "g" abbreviation throughout (not "grams" spelled out mid-sentence)

• Tesla: standardized to "T" abbreviation after first use

We believe the revised manuscript comprehensively addresses all reviewer concerns. We hope the revised version meets the standards of PLOS ONE and will be suitable for publication.

Yours sincerely,

Zdenek Starek, MD, PhD, on behalf of all authors

Attachment Submitted filename: Reviewer_com_answer_V5.docx

# Editor report: Decision Letter 1

**DOI:** 10.1371/journal.pone.0357588.r003

**Authors:** Ibrahim Marai

19 Aug 2026

<p>In Vitro Contact Force Evaluation of Robotic Magnetic Navigation Catheters; Head to Head Comparison of Different Catheters and Different Settings

PONE-D-26-14424R1

Dear Dr. Zdenek Starek

We’re pleased to inform you that your manuscript has been judged scientifically suitable for publication and will be formally accepted for publication once it meets all outstanding technical requirements.

Within one week, you’ll receive an e-mail detailing the required amendments. When these have been addressed, you’ll receive a formal acceptance letter and your manuscript will be scheduled for publication.

An invoice will be generated when your article is formally accepted. Please note, if your institution has a publishing partnership with PLOS and your article meets the relevant criteria, all or part of your publication costs will be covered. Please make sure your user information is up-to-date by logging into Editorial Manager at Editorial Manager® and clicking the ‘Update My Information' link at the top of the page. For questions related to billing, please contact billing support.

If your institution or institutions have a press office, please notify them about your upcoming paper to help maximize its impact. If they’ll be preparing press materials, please inform our press team as soon as possible -- no later than 48 hours after receiving the formal acceptance. Your manuscript will remain under strict press embargo until 2 pm Eastern Time on the date of publication. For more information, please contact onepress@plos.org.

Kind regards,

Ibrahim Marai, MD

Academic Editor

PLOS One

# Editor report: Acceptance letter

**DOI:** 10.1371/journal.pone.0357588.r004

**Authors:** Ibrahim Marai

PONE-D-26-14424R1

PLOS One

Dear Dr. Starek,

I'm pleased to inform you that your manuscript has been deemed suitable for publication in PLOS One. Congratulations! Your manuscript is now being handed over to our production team.

At this stage, our production department will prepare your paper for publication. This includes ensuring the following:

* All references, tables, and figures are properly cited

* All relevant supporting information is included in the manuscript submission,

* There are no issues that prevent the paper from being properly typeset

You will receive further instructions from the production team, including instructions on how to review your proof when it is ready. Please keep in mind that we are working through a large volume of accepted articles, so please give us a few days to review your paper and let you know the next and final steps.

Lastly, if your institution or institutions have a press office, please let them know about your upcoming paper now to help maximize its impact. If they'll be preparing press materials, please inform our press team within the next 48 hours. Your manuscript will remain under strict press embargo until 2 pm Eastern Time on the date of publication. For more information, please contact onepress@plos.org.

You will receive an invoice from PLOS for your publication fee after your manuscript has reached the completed accept phase. If you receive an email requesting payment before acceptance or for any other service, this may be a phishing scheme. Learn how to identify phishing emails and protect your accounts at https://explore.plos.org/phishing.

If we can help with anything else, please email us at customercare@plos.org.

Thank you for submitting your work to PLOS One and supporting open access.

Kind regards,

PLOS One Editorial Office Staff

on behalf of

Dr. Ibrahim Marai

Academic Editor

PLOS One
